# Community Based Assessment of Behavior and Awareness of Risk Factors of Cystic Echinococcosis in Major Cities of Pakistan: A One Health Perspective

**DOI:** 10.3389/fpubh.2021.648900

**Published:** 2021-06-04

**Authors:** Aisha Khan, Haroon Ahmed, Shaheera Amjad, Muhammad Sohail Afzal, Waseem Haider, Sami Simsek, Mudassar Rashid Khawaja, Danish Hassan Khan, Shumaila Naz, Anna Durrance-Bagale, Rana Muhammad Kamran Shabbir, Raja Zoq Ul Arfeen, Shahzad Ali, Jianping Cao

**Affiliations:** ^1^Department of Biosciences, COMSATS University Islamabad, Islamabad, Pakistan; ^2^Key Laboratory of Parasite and Vector Biology, National Health Commission of the People's Republic of China, Shanghai, China; ^3^Department of Lifesciences, School of Science, University of Management and Technology, Lahore, Pakistan; ^4^Department of Parasitology, Firat University, Elâziğ, Turkey; ^5^Department of Economics, COMSATS University Islamabad, Islamabad, Pakistan; ^6^Department of Biological Sciences, National University of Medical Sciences, Rawalpindi, Pakistan; ^7^Faculty of Public Health and Policy, London School of Hygiene and Tropical Medicine, London, United Kingdom; ^8^School of Food and Agricultural Sciences, University of Management and Technology (UMT), Lahore, Pakistan; ^9^Department of Wildlife and Ecology, University of Veterinary and Animal Sciences, Lahore, Pakistan; ^10^National Institute of Parasitic Diseases, Chinese Center for Disease Control and Prevention, Shanghai, China; ^11^The School of Global Health, Chinese Center for Tropical Diseases Research, Shanghai Jiao Tong University School of Medicine, Shanghai, China; ^12^WHO Collaborating Centre for Tropical Diseases, Shanghai, China

**Keywords:** cystic echinococcosis, community perception, one health concept, risk factors, Pakistan

## Abstract

**Background:** The parasitic disease, cystic echinococcosis (CE), is a serious health problem in Pakistan. Risk of disease transmission is increased by economic and political instability, poor living conditions, and limited awareness of hygienic practices. The current study aimed to investigate the community perception and awareness regarding the risk factors of CE in Pakistan, from a One Health perspective.

**Methods:** We conducted a community-based survey involving 454 participants in the major cities of Pakistan. Quantitative data based on knowledge, attitude, and practices (KAP), the One Health concept, risk factors, and community perception of CE among the general population of the major cities of Pakistan were collected. The questions included those related to knowledge, attitude, practices, One Health concept, risk factors, and community perception. The Chi-squared test was applied to determine the associations regarding KAPs across socio-demographic parameters.

**Results:** KAPs had no significant associations with sociodemographic aspects such as age, sex, religion, ethnicity, education, marital status, occupation, or financial status of the participants. The findings indicated a lack of awareness about CE among the participants. Respondents were unaware of the risk factors and the One Health concept of CE. However, the community attitude and perception were positive toward the control of CE.

**Conclusion:** Illiteracy, deficient sanitation systems and lack of awareness are the contributing factors to CE in Pakistan. It is necessary to make the community aware regarding CE and its importance. Increasing this awareness represents an important step toward the eradication and control of CE.

## Introduction

Zoonotic diseases spread between animals (usually vertebrates) and humans via direct or indirect contact (1), and represent about one-fifth of parasitic infections in nature (2). Echinococcosis is one of the most significant and widespread chronic diseases worldwide (3, 4). It is caused by taeniid *Echinococcus* spp. tapeworms at their larval stages, the life cycle of which involves two vertebrate hosts: definitive and intermediate (5). Humans are occasional intermediate hosts, and are infected via ingestion of contaminated food or water, or through contact with infected definitive hosts (6). *Echinococcus multilocularis* (causing alveolar echinococcosis) and *E. granulosus sensu lato* (causing cystic echinococcosis) are of major public health importance (7).

Cystic echinococcosis (CE) is a serious public health problem around the world (8) and is listed as one of the 20 neglected tropical diseases (NTDs) in the Neglected Zoonotic Diseases (NZDs) sub-group by the World Health Organization (WHO) (9, 10). CE is more common in sheep-raising countries (8, 11–14). Human CE has the highest prevalence in the eastern part of the Mediterranean region, southern and eastern Europe, and the least half of South America, Northern Africa, Australia, Russia, Western China, Siberia, and in Central Asia, where approximately 2–3 million people are infected and 200,000 new cases are reported annually (9, 14, 15). Central Asia, including Afghanistan, Kazakhstan, Kyrgyzstan, Iran, Tajikistan, Turkmenistan, Mongolia, Uzbekistan, Western China, and Pakistan, is a highly endemic region for CE, with ~58% of the population at being at risk (16).

Pakistan is an agricultural country, in which about 47% of the population are involved in agriculture. Livestock, the backbone of the agricultural sector, has a significant role in the economy of Pakistan, contributing almost 56.3% of the agricultural value and 11% of the GDP (17, 18). However, parasitic infections (including echinococcosis) cause economic losses of around 26.5 million rupees or $354000US annually to the livestock sector (19). Many reasons, including insufficiently equipped abattoirs located in the vicinity of residential areas, the proximity of animals (especially dogs and livestock) to humans, poor public awareness, and unhygienic lifestyles favor the lifecycle of *E. granulosus* (20).

Human health is strongly related to a country's socioeconomic status. However, various aspects of socioeconomic status (education, ethnicity, and financial resources) are disproportionately linked to health. Some aspects promote health, some are aspects are promoted by health, some are mutually determined with health, and some fall in all three categories (21). Pakistan is a country with low socioeconomic status, where aspects of socioeconomic status generally have a negative impact on health, because it is highly populated (around 200 million inhabitants) with poor living standards, and most of the population lives in underdeveloped, rural settings. Lifestyle in these areas, where humans and animals often share the same residences, and poor health and hygiene practices are followed, is a major risk for disease transmission (22). According to a Pakistan economic survey, 38% of the population was declared poor during in 2015/2016, with 41% in rural areas and 32% in urban areas. Only around 5% of households have access to clean water, proper sanitation, electricity, and cooking fuel. *E. granulosus s.s* (G1–G3 genotypes) has already been reported in livestock in Pakistan, and all factors are likely to contribute to its further spread among the population (23).

Over time, the livestock sub-sector in Pakistan has surpassed the crops sub-sector as the primary contributor to agriculture. During 2019-20, livestock contributed 60.6% to overall agriculture and 11.7% to GDP. The value of the livestock sector can be gauged by the fact that it accounts for around 3.1% of total exports and provides 35-40% of income for more than 8 million rural households. Goats (78.2 million), cattle (49.6 million), buffalo (41.2 million), and sheep make up the majority of Pakistan's livestock (31.2 million). The goat population in Pakistan ranks third in the world, after India and China, in livestock farming. Sheep and goats contribute significantly to the economy of the country by providing milk, meat, beef, and hides. Agriculture contributes 19.3% to Pakistan's GDP and employs 42.3% of the population. Despite a large population of sheep and goats, Pakistan's low volume ruminant production is severely hampered by a number of factors, including a lack of acaricides (24).

Few studies have examined the drivers of CE. Between 1990 and 2018, 15 retrospective survey-based studies and 19 case studies reported 1,611 cases of CE in Pakistan. The absence of a surveillance system or a national database to identify and record CE cases has resulted in a substantial data gap (16). As an infectious disease, the success of CE prevention and control programmes depends on community cooperation. Understanding all the disease-related aspects is an important determinant of community participation in programme implementation. In the present study, we investigated community knowledge, attitudes, and the One Health approach toward CE in Pakistan, particularly related to health and risk factors.

## Materials and Methods

### Study Design, Sampling and Selection Criteria

**Figure 1:**
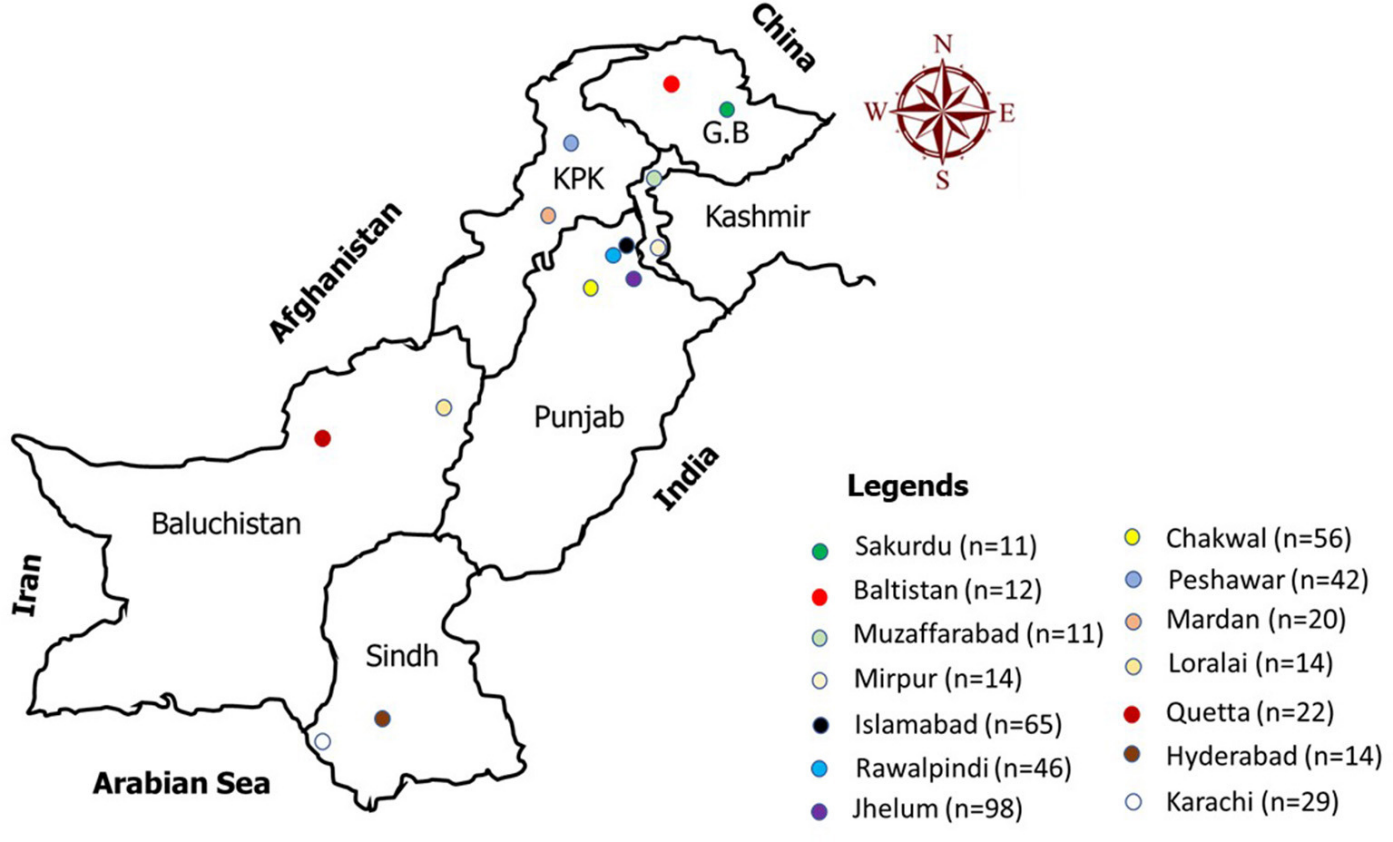
Map of Pakistan with multiple dots depicting the sampling sites where the data were collected.

Pakistan is one of the most suitable countries for studying CE because the livestock sector is a major contributor to the economy. Many geographical and demographical features also promote the onset and spread of CE. This study aimed to evaluate the level of awareness of CE among residents of Pakistan; therefore, we interviewed people in major cities, including Rawalpindi, Islamabad, Chakwal, Jhelum (including Kalar Kahar), Quetta, Karachi, Hyderabad, Lahore, Peshawar, and Northern Areas (Figure 1). Urban and rural areas around or within these cities were included. We used convenience sampling to select the participants. Pakistan is an ethnically diverse country with many different communities, e.g., Punjabi, Pathan, and Kashmiri; therefore, a diverse sample is possible even in one city. Individuals with or without any kind of animal association and of any occupational and educational background were considered for sampling. All participants were aged at least 15 years old.

A total of 454 people participated in the study (Table 1). Hospital staff; those in educational institutions, markets, and homes; and transport passengers were surveyed using face to face interview.

**Table 1:** Sociodemographic characteristics of the participants.

**Variables**	**Participants** ** (No.)**	**Frequency** ** (%)**
**Provinces**		
Punjab	265	58.4
Sindh	43	9.47
Baluchistan	36	7.93
KPK	62	13.7
AJK	25	5.51
GB	23	5.07
**Age**		
15–30	325	71.6
31–45	83	18.3
46–60	37	8.1
61–75	9	2.0
**Sex**		
Female	256	56.4
Male	198	43.6
**Religion**		
Muslim	448	98.77
Christian	3	0.7
Hindu	2	0.4
Sikh	1	0.2
**Ethnicity**		
Punjabi	239	52.6
Sindhi	6	1.3
Baluchi	11	2.4
Pathan	62	13.7
Kashmiri	13	2.9
Balti	6	1.3
Others	117	25.8
**Education**		
Post-secondary	403	88.8
Secondary level	21	4.6
Primary level	16	3.5
No formal education	14	3.1
**Marital Status**		
Single	319	70.3
Married	135	29.7
**Occupation**		
Butchers	1	0.2
Farmers	34	7.5
Livestock keepers	14	3.1
**Other professions**		
Student	217	47.8
Shop keeper	80	17.6
Teacher	35	7.7
Business	6	1.3
No work	5	1.1
Engineer	4	0.9
Housewife	4	0.9
Timber Merchants	3	0.7
Driver	3	0.7
Zoologist	1	0.2
Unemployed	1	0.2
Self employed business owner	1	0.2
Sales man	1	0.2
Retired	2	0.4
Restaurant business	26	5.7
Lab technologist	2	0.4
Service provider	2	0.4
Health	1	0.2
Free lancer	2	0.4
Supplier	1	0.2
Doctor	1	0.2
Dentist	3	0.7
Designer	1	0.2
Computer scientist	1	0.2
Biotechnologist	1	0.2
Administration	1	0.2
**Income**		
30,000 & below	62	13.7
31,000–60,000	121	26.7
61,000–90,000	91	20.0
90,000 above	180	39.6

### Community Questionnaire Survey

A community-based, cross-sectional study was designed to collect the data. An easy descriptive questionnaire was designed for both rural and urban participants. For participants not able to read or write, face-to-face interviews were conducted using the same questionnaire to obtain the data.

The questionnaire was divided into six major categories:

(1) Knowledge regarding CE, such as general awareness of the disease and its mode of transmission.
(2) Attitude, such as views on symptoms, treatment, and diagnosis.
(3) Practices related to the disease, such as the feed given to dogs, hand washing, and proximity to dogs.
(4) Risk factors regarding CE in the surveyed areas, such as the consumption of contaminated food and water, and slaughtering systems
(5) Social, political, and economic instability commonly observed in the surveyed areas.
(6) Questions related to the One Health concept (animal-environment-human) of CE and the community perception of prevention and control.

All these questions had either a Yes or No answer (see Supplementary Material).

### Data Management and Statistical Analysis

This was a multivariate analysis, including dependent and independent variables.

### Dependent Variables

These included: (1) knowledge about CE, (2) attitude toward treatment of infection or exposure to animals, (3) practices and factors associated with the spread of CE, (4) the One Health concept related to CE, (5) risk factors of CE, and (6) community perception in general toward CE.

### Independent Variables

These were sex, age, occupation, level of education, religion, ethnicity, marital status, and income.

A database was established using MS Excel (Microsoft, Redmond, WA, USA) and then analyzed statistically using SPSS (v. 23 Version; IBM Corp., Armonk, NY, USA). Bivariate correlation was checked with the help of the Chi-squared test, where one dependent variable was weighed against one independent variable. The relationships of the different sociodemographic factors to knowledge, attitudes, practices, the One Health concept, risk factors, and community perception were analyzed. Statistically significant results were recorded at *p* < 0.05.

## Results

### Sociodemographic Background

Men (*n* = 198; 44%) and women (*n* = 256; 56%) ranged in age from 16 to 75 years. Most participants were of Punjabi ethnicity (53%), followed by Pathans (14%), Kashmiri (3%), Baloch (2%), Sindhi and Balti (1%), with 26% from other ethnicities. About 11% worked directly or indirectly with livestock, including farmers (8%), livestock owners (3%), and butchers (0.2%). Approximately 89% of the participants had other professions. Regarding the level of education, 5% were educated up to secondary level and 3% had no formal education (Table 1).

### KAP Analysis

Regarding knowledge, 68% (309/454) of participants had never heard about zoonoses and 80% (361/454) were unaware of CE. The majority had never seen the disease in any individual or animal. Approximately 90% (410/454) of the respondents did not know that they could get infected with the disease. The attitude of participants was quite positive: 62% (283/454) would accept CE inspection, treatment, or surgery (if required). Stray dogs were reported by 58% (265/454) of participants; 85% (385/454) of participants washed their hands after handling cattle and 90% (407/454) washed their hands before eating. Only 58% (262/454) reported that slaughter areas were well-managed, while 64% (288/454) reported meat inspection before consumption (Table 2).

**Table 2:** Knowledge, attitude, and practices (KAPs) toward CE among the study respondents.

**Variables**	**Characteristics**	**Participants (No.)**	**Frequency (%)**
Ever heard about zoonotic disease?	Yes	145	31.9
	No	309	68.1
Ever heard about echinococcosis?	Yes	93	20.5
	No	361	79.5
At risk of developing echinococcosis?	Yes	44	9.7
	No	410	90.3
Became infected by association with dogs?	Yes	245	54
	No	209	46
Would you receive disease inspection/ treatment/surgery?	Yes	283	62.3
	No	171	37.7
Do you own dog(s)?	Yes	56	12.3
	No	398	87.7
Stray dogs in your area?	Yes	265	58.4
	No	189	41.6
Are dogs fed slaughter waste?	Yes	125	27.5
	No	329	72.5
Do you wash your hands before eating food?Do you wash your hands after handling animals?	Yes	407	89.6
	No	47	10.4
	Yes	385	84.8
	No	69	15.2
Do you inspect meat?	Yes	288	63.4
	No	166	36.6
Are the slaughter areas clean and well-managed?	Yes	192	42.3
	No	262	57.7

### The One Health Concept of Cystic Echinococcosis

Despite low awareness of the disease, the response of participants toward the concept of One Health was positive. For all seven questions about One Health, most stated that all the suggested steps were important to ensure a healthy environment for animals and thus promote human health. Highest support (94%) was seen for the requirement of proper treatment facilities, followed by awareness and vaccination campaigns (91%). There was a positive response for the suggestion of proper waste disposal systems (93%) and food inspection (92%; Table 3).

**Table 3:** Representation of one health concept of CE across different variables.

**Variable**	**Characteristics**	**Participants (No.)**	**Frequency (%)**
Humans linked to animals, environment?	Yes	413	91
	No	41	9
Vaccination campaigns required?	Yes	415	91.4
	No	39	8.6
Proper treatment facilities needed?	Yes	427	94.1
	No	27	5.9
Need for disposal systems?	Yes	424	93.4
	No	30	6.6
Diet should be inspected properly?	Yes	416	91.6
	No	38	8.4
Awareness of the impact of the environment?	Yes	417	91.9
	No	37	8.1
Economic stability to favor/improve health?	Yes	408	89.9
	No	46	10.1

### Risk Factors of Cystic Echinococcosis

Regarding the risk factors surveyed, most (86%) of the participants considered lack of awareness as an important risk factor for the disease. Others identified unchecked systems of animal keeping (80%), contaminated water/food consumption (74%), and social, political, and economic instability (82%; Table 4).

**Table 4:** Representation of Risk factors of CE across different variables.

**Variable**	**Characteristics**	**Participants (No.)**	**Frequency (%)**
Social, political, economic instability?	Yes	372	81.9
	No	82	18.1
Unchecked systems of animal keeping?	Yes	363	80
	No	91	20
Lack of awareness?	Yes	389	85.7
	No	65	14.3
Exposure to dog feces?	Yes	308	67.8
	No Don't know	1442	31.7 0.4
Contaminated food/water consumption?	Yes	336	74
	No	118	26
Asymptomatic disease?	Yes	279	61.5
	No Don't know	16114	35.5 3.1

### Community Perceptions of Cystic Echinococcosis

We suggested 10 preventive or precautionary measures people could take to reduce, avoid, or eliminate CE. For each measure, two options were provided: one in favor of the measure and the other against it. Only 17% of participants favored the option of killing all dogs, while 47.6% thought only stray dogs should be killed. Sixty two point eight percentage thought reducing dogs' access to slaughter areas would be enough to eliminate the disease. Almost 50:50 ratios were found for prevention and treatment, with respondents considering each to be a disease control measure (Table 5).

**Table 5:** Representation of Community perception about CE across different variables.

**Variable**	**Characteristics**	**Participants (No.)**	**Frequency (%)**
Kill all dogs?	Favor	78	17.2
	Against	376	82.8
Kill stray dogs only?	Favor	216	47.6
	Against	238	52.4
Stop feeding dogs with sheep cysts?	Favor	254	55.9
	Against	200	44.1
Feeding dogs personally?	Favor	298	65.6
	Against	156	34.4
Prevention vs. treatment?	Favor	235	51.8
	Against	219	48.2
Bury/burn infected organs?	Favor	292	64.3
	Against	162	35.7
Stop owning dogs?	Favor	242	53.3
	Against	212	46.7
Stop throwing away carcasses?	Favor	323	71.1
	Against	131	28.9
Replace sheep with goats?	Favor	277	61
	Against	177	39
Reduce dogs' access to slaughter areas?	Favor	285	62.8
	Against	169	37.2

### Analysis of the One Health Concept, Risk Factors, and Community Perception of CE Based on Various Sociodemographic Factors

We analyzed all data with reference to sociodemographic factors to ascertain whether CE awareness and views vary according to age, sex, ethnicity, religion, education, marital status, occupation, and income (Tables 6–8). While testing for the One Health concept, Q3 had significant associations (*p* < 0.05) with sex and religious orientation. The response to Q1 was different in various ethnic groups. The responses to Q2, Q6, and Q7 (Table 6) varied by different educational backgrounds. For the risk factors (Table 7), different age groups had different responses to Q4 (*p* < 0.05), while the different sexes had significantly different responses to Q2 and Q6. The various ethnic groups had different responses to Q2, Q3, Q4, Q5, and Q6. People with various educational levels responded differently to Q, Q4, Q5, and Q6 while people with different occupations responded similarly except for Q3 and Q4. Income levels did not seem to have much influence on people perception of risk. In community perception (Table 8), marital status had a significant effect on opinion (response to Q1, Q2, Q8, and Q10) while the responses to Q10 were associated with age, education and ethnicity, Q3 was associated with sex and Q4 was associated with education (*p* < 0.05).

**Table 6:** Representation of Sociodemographic factors across One Health concept of CE questions.

**Sociodemographic Factors**		**One Health (%)**													
**Variables**	**Features**	**Q1**		**Q2**		**Q3**		**Q4**		**Q5**		**Q6**		**Q7**	
		**Yes**	**No**	**Yes**	**No**	**Yes**	**No**	**Yes**	**No**	**Yes**	**No**	**Yes**	**No**	**Yes**	**No**
Age	15–30	295 (71.4)	30 (73.2)	297 (71.6)	28 (71.8)	306 (71.7)	19 (70.4)	304 (71.7)	21 (70)	298 (71.6)	27 (71.1)	300 (71.9)	25 (67.6)	293 (71.8)	32 (69.6)
	31–45	77 (18.6)	6 (14.6)	79 (19)	4 (10.3)	79 (18.5)	4 (14.8)	78 (18.4)	5 (16.7)	76 (18.3)	7 (18.4)	77 (18.5)	6 (16.2)	76 (18.6)	7 (15.2)
	46–60	33 (8.0)	4 (9.8)	31 (7.5)	6 (15.4)	34 (8.0)	3 (11.1)	35 (8.3)	2 (6.7)	35 (8.4)	2 (5.3)	32 (7.7)	5 (13.5)	31 (7.6)	6 (13.0)
	61–75	8 (1.9)	1 (2.4)	8 (1.9)	1 (2.6)	8 (1.9)	1 (3.7)	7 (1.7)	2 (6.7)	7 (1.7)	2 (5.3)	8 (1.9)	1 (2.7)	8 (2.0)	1(2.2)
Statistical analysis		χ2 = 0.534, df = 3, *P* = 0.911		χ2 = 4.316, d f= 3, *P* = 0.229		χ2 = 0.933, df = 3, *P* = 0.818		χ2 = 3.699, df = 3, *P* = 0.296		χ2 = 0.2678, df = 3, *P* = 0.444		χ2 = 1.712, df = 3, *P* = 0.634		χ2 = 1.806, df = 3, *P* = 0.614	
Sex	Male	184 (44.6)	14 (34.1)	184 (44.3)	14 (35.9)	192 (45.0)	6 (22.2)	185 (43.6)	13(43.3)	183 (44.0)	15 (39.5)	198 (43.6)	16 (43.2)	178 (43.6)	20 (43.5)
	Female	229 (55.4)	27 (65.9)	231 (55.7)	25 (64.1)	235 (55.0)	21 (77.8)	239 (56.4)	17 (56.7)	233 (56.0)	23 (60.5)	235 (56.4)	21 (56.8)	230 (56.4)	26 (56.5)
Statistical analysis		χ2 = 1.642, df = 1, *P* = 0.200		χ2 = 1.033, df = 1, *P* = 0.310		χ2 = 5.341, df = 1, *P* = 0.021		χ2 = 0.001, df = 1, *P* = 0.975		χ2 = 0.289, df = 1, *P* = 0.591		χ2 = 0.002, df = 1, *P* = 0.962		χ2 = 0.000, df = 1, *P* = 0.985	
Religion	Islam	407 (98.5)	41 (100)	409 (98.6)	39 (100)	423 (99.1)	25 (92.6)	418 (98.6)	30 (100)	410 (98.6)	38 (100)	411 (98.6)	37 (100)	402 (98.5)	46 (100)
	Christianity	3 (0.7)	0 (0.0)	3 (0.7)	0 (0.0)	1 (0.2)	2 (7.4)	3 (0.7)	0 (0.0)	3 (0.7)	0 (0.0)	3 (0.7)	0 (0.0)	3 (0.7)	0 (0.0)
	Hindu	2 (0.5)	0 (0.0)	2 (0.5)	0 (0.0)	2 (0.5)	0 (0.0)	2 (0.5)	0 (0.0)	2 (0.5)	0 (0.0)	2 (0.5)	0 (0.0)	2 (0.5)	0 (0.0)
	Sikhism	1 (0.2)	0 (0.0)	1 (0.2)	0 (0.0)	1 (0.2)	0 (0.0)	1 (0.2)	0 (0.0)	1 (0.2)	0 (0.0)	1 (0.2)	0 (0.0)	1 (0.2)	0 (0.0)
Statistical analysis		χ2 = 0.604, df = 3, *P* = 0.896		χ2 = 0.571, df = 3, *P* = 0.903		χ2 = 20.072, df = 3, *P* = 0.000		χ2 = 0.430, df = 3, *P* = 0.934		χ2 = 0.555, df = 3, *P* = 0.907		χ2 = 0.540, df = 3, *P* = 0.910		χ2 = 0.686, df = 3, *P* = 0.877	
Ethnicity	Punjabi	222 (53.8)	17 (41.5)	220 (53.0)	19 (48.7)	228 (53.4)	11 (40.7)	227 (53.5)	12 (40.0)	218 (52.4)	21 (55.3)	220 (52.8)	19 (51.4)	121 (52.0)	27 (58.7)
	Sindhi	6 (1.5)	0 (0.0)	6 (1.4)	0 (0.0)	6 (1.4)	0 (0.0)	6 (1.4)	0 (0.0)	6 (1.4)	0 (0.0)	6 (1.4)	0 (0.0)	6 (1.5)	0 (0.0)
	Balochi	10 (2.4)	1 (2.4)	10 (2.4)	1 (2.6)	10 (2.3)	1 (3.7)	10 (2.4)	1 (3.3)	11 (2.6)	0 (0.0)	11 (2.6)	0 (0.0)	10 (2.5)	1 (2.2)
	Pathan	60 (14.5)	2 (4.9)	60 (14.5)	2 (5.1)	61 (14.3)	1 (3.7)	57 (13.4)	5 (16.7)	59 (14.2)	3 (7.9)	60 (14.4)	2 (5.4)	60 (14.7)	2 (4.3)
	Balti	4 (1.0)	2 (4.9)	5 (1.2)	1 (2.6)	6 (1.4)	0 (0.0)	3 (0.7)	3 (10.0)	5 (1.2)	1 (2.6)	6 (1.4)	0 (0.0)	4 (1.0)	2 (4.3)
	Kashmiri	13 (3.1)	0 (0.0)	13 (3.1)	0 (0.0)	11 (2.6)	2 (7.4)	13 (3.1)	0 (0.0)	13 (3.1)	0 (0.0)	12 (2.9)	1 (2.7)	13 (3.2)	0 (0.0)
	Others	98 (23.7)	10 (46.3)	101 (24.3)	16 (41.0)	105 (24.6)	12 (44.4)	108 (25.5)	9 (30.0)	104 (25.0)	13 (34.2)	102 (24.5)	15 (40.5)	103 (25.2)	14 (30.4)
Statistical analysis		χ2 = 17.213, df = 6, *P* = 0.009		χ2 = 8.537, df = 6, *P* = 0.201		χ2 = 9.762, df = 6, *P* = 0.135		χ2 = 21.172, df = 6, *P* = 0.002		χ2 = 5.874, df = 6, *P* = 0.483		χ2 = 7.475, df = 6, *P* = 0.279		χ2 = 9.738, df = 6, *P* = 0.136	
Education	No formal education	13 (3.1)	1 (2.4)	14 (3.4)	0 (0.0)	14 (3.3)	0 (0.0)	14 (3.3)	0 (0.0)	14 (3.4)	0 (0.00)	14 (3.4)	0 (0.0)	14 (3.4)	0 (0.0)
	Primary	14 (3.4)	2 (4.9)	14 (3.4)	2 (5.1)	15 (3.5)	1 (3.7)	14 (3.3)	2 (6.7)	15 (3.6)	1 (2.6)	15 (3.6)	1 (2.7)	14 (3.4)	2 (4.3)
	Secondary	16 (3.9)	5 (12.2)	15 (3.6)	6 (15.4)	19 (4.4)	2 (7.4)	18 (4.2)	3 (10.0)	17 (4.1)	4 (10.5)	15 (3.6)	6 (16.2)	15 (3.7)	6 (13.0)
	Post-secondary	370 (89.6)	33 (80.5)	372 (89.6)	31 (79.5)	379 (88.8)	24 (88.9)	378 (89.2)	25 (83.3)	370 (88.9)	33 (86.8)	373 (89.4)	30 (81.1)	365 (89.5)	38 (82.6)
Statistical analysis		χ2 = 6.226, df = 3, *P* = 0.101		χ2 = 12.718, df = 3, *P* = 0.005		χ2 = 1.368, df = 3, *P* = 0.713		χ2 = 4.003, df = 3, *P* = 0.261		χ2 = 4.512, df = 3, *P* = 0.211		χ2 = 13.287, df = 3, *P* = 0.004		χ2 = 9.737, df = 3, *P* = 0.021	
Marital status	Single	291 (70.5)	28 (68.3)	292 (70.4)	27 (69.2)	302 (70.7)	17 (63.0)	300 (70.8)	19 (63.3)	292 (70.2)	27 (71.1)	295 (70.7)	24 (64.9)	288 (70.6)	31 (67.4)
	Married	122 (29.5)	13 (31.7)	123 (29.6)	12 (30.8)	125 (29.3)	10 (37.0)	124 (29.9)	11 (36.7)	124 (29.8)	11 (28.9)	122 (29.3)	13 (35.1)	120 (29.4)	15 (32.6)
Statistical analysis		χ2 = 0.84, df = 1, *P* = 0.772		χ2 = 0.022, df = 1, *P* = 0.883		χ2 = 0.732, df = 1, *P* = 0.392		χ2 = 0.739, df = 1, *P* = 0.390		χ2 = 0.012, df = 1, *P* = 0.912		χ2 = 0.562, df = 1, *P* = 0.453		χ2 = 0.202, df = 1, *P* = 0.653	
Occupation	Butchers	1 (0.2)	0 (0.0)	1 (0.2)	0 (0.0)	1 (0.2)	0 (0.0)	1 (0.2)	0 (0.0)	1 (0.2)	0 (0.0)	1 (0.2)	0 (0.0)	1 (0.2)	0 (0.0)
	Farmers	31 (7.5)	3 (7.3)	32 (7.7)	2 (5.1)	34 (8.0)	0 (0.0)	33 (7.8)	1 (3.3)	32 (7.7)	2 (5.3)	32 (7.7)	2 (5.4)	28 (6.9)	6 (13.0)
	Livestock Keepers	12 (2.9)	2 (4.9)	12 (2.9)	2 (5.1)	12 (2.8)	2 (7.4)	13 (3.1)	1 (3.3)	13 (3.1)	1 (2.6)	14 (3.4)	0 (0.0)	1.3 (3.2)	1 (2.2)
	Others	369 (89.3)	36 (87.8)	370 (89.2)	35 (89.7)	380 (89.0)	25 (92.6)	377 (88.9)	28 (93.3)	370 (88.9)	35 (92.1)	370 (88.7)	35 (94.6)	366 (89.7)	39 (84.8)
Statistical analysis		χ2 = 0.582, df = 3, *P* = 0.901		χ2 = 0.991, df = 3, *P* = 0.803		χ2 = 3.990, df = 3, *P* = 0.263		χ2 = 0.879, df = 3, *P* = 0.830		χ2 = 0.432, df = 3, *P* = 0.934		χ2 = 1.696, df = 3, *P* = 0.638		χ2 = 2.471, df = 3, *P* = 0.481	
Income	Below 30,000	55 (13.3)	7 (17.1)	55 (13.3)	7 (17.9)	59 (13.8)	3 (11.1)	60 (14.2)	2 (6.7)	59 (14.2)	3 (7.9)	56 (13.4)	6 (16.2)	53 (13.0)	9 (19.6)
	31,000–60,000	111 (26.9)	10 (24.4)	111 (26.7)	10 (25.6)	113 (26.5)	8 (29.6)	115 (27.1)	6 (20.0)	113 (27.2)	8 (21.1)	112 (26.9)	9 (24.3)	111 (27.2)	10 (21.7)
	61,000–90,000	83 (20.1)	8 (19.5)	83 (20.0)	8 (20.5)	87 (20.4)	4 (14.8)	84 (19.8)	7 (23.3)	80 (19.2)	11 (28.9)	86 (20.6)	5 (13.5)	83 (20.0)	8 (17.4)
	Above 90,000	164 (39.7)	16 (39.0)	166 (40.0)	14 (35.9)	168 (39.3)	12 (44.4)	165 (38.9)	15 (50.0)	164 (39.4)	16 (42.1)	163 (39.1)	17 (45.9)	161 (39.5)	19 (41.3)
Statistical analysis		χ2 = 0.483, df = 3, *P* = 0.923		χ2 = 0.748, df = 3, *P* = 0.862		χ2 = 0.790, df = 3, *P* = 0.852		χ2 = 2.724, df = 3, *P* = 0.436		χ2 = 3.199, df = 3, *P* = 0.362		χ2 = 1.535, df = 3, *P* = 0.674		χ2 = 1.987, df = 3, *P* = 0.575	

*Q1, Do you agree that the health of humans is linked to health of animals and the environment?*

*Q2, Are vaccination campaigns for animals and humans are required?*

*Q3, Are proper treatment facilities needed?*

*Q4, Is there a need for proper disposal and sewage systems?*

*Q5, Should the diet of people as well as animals should be inspected properly?*

*Q6, Are you aware of the impact of the environment on humans and animals?*

*Q7, Does economic stability to favor/improve health?*

**Table 7:** Representation of Sociodemographic factors based on risk factors of CE questions.

**Sociodemographic factors**		**Risk Factors (%)**											
**Variables**	**Features**	**Q1**		**Q2**		**Q3**		**Q4**		**Q5**		**Q6**	
		**Yes**	**No**	**Yes**	**No**	**Yes**	**No**	**Yes**	**No**	**Yes**	**No**	**Yes**	**No**
Age	15–30	266 (71.5)	59 (72)	225 (70.2)	70 (76.9)	274 (70.4)	51 (78.5)	21 (69.8)	110 (76.4)	231 (68.8)	94 (79.9)	196 (70.3)	118 (73.3)
	31–45	68 (18.3)	15 (18.3)	67 (18.5)	16 (17.6)	75 (19.3)	8 (12.3)	63 (20.5)	20 (13.9)	69 (20.5)	14 (11.9)	55 (19.7)	27 (16.8)
	46–60	31 (8.3)	6 (7.3)	33 (9.1)	4 (4.4)	32 (8.2)	5 (7.7)	26 (8.4)	10 (6.9)	29 (8.6)	8 (6.8)	23 (8.2)	13 (8.1)
	61–75	7 (1.9)	2 (2.4)	8 (2.2)	1 (1.1)	8 (2.1)	1 (1.5)	4 (1.3)	2 (2.8)	7 (2.1)	2 (1.7)	5 (1.8)	3 (1.9)
Statistical analysis		χ2 = 0.192, df = 3, *P* = 0.979		χ2 = 2.900, df = 3, *P* = 0.407		χ2 = 2.077, df = 3, *P* = 0.557		χ2 = 33.748, df = 6, *P* = 0.000		χ2 = 5.478, df = 3, *P* = 0.140		χ2 = 3.659, df = 6, *P* = 0.723	
Sex	Male	165 (44.4)	33 (40.2)	197 (54.3)	59 (64.8)	218 (56.0)	38 (58.5)	170 (55.2)	86 (59.7)	181 (53.9)	75 (63.6)	150 (53.8)	103 (64.0)
	Female	207 (55.6)	49 (59.8)	166 (45.7)	32 (35.2)	171 (44.0)	27 (41.5)	138 (44.8)	58 (40.3)	155 (46.1)	43 (36.4)	129 (46.2)	58 (36.0)
Statistical analysis		χ2 = 0.462, df = 1, *P* = 0.497		χ2 = 3.303, df = 1, *P* = 0.069		χ2 = 0.133, df = 1, *P* = 0.716		χ2 = 3.415, df = 2, *P* = 0.181		χ2 = 3.335, df = 1, *P* = 0.068		χ2 = 11.508, df = 2, *P* = 0.003	
Religion	Islam	366 (98.4)	82 (100)	357 (98.3)	91 (100)	384 (98.7)	64 (98.5)	303 (98.4)	143 (99.3)	333 (99.1)	115 (97.5)	276 (98.9)	158 (98.1)
	Christianity	3 (0.8)	0 (0.0)	3 (0.8)	0 (0.0)	2 (0.5)	1 (1.5)	2 (0.6)	1 (0.7)	0 (0.0)	3 (2.5)	0 (0.0)	3 (1.9)
	Hindu	2 (0.5)	0 (0.0)	2 (0.6)	0 (0.0)	2 (0.5)	0 (0.0)	2 (0.6)	0 (0.0)	2 (0.6)	0 (0.0)	2 (0.7)	0 (0.0)
	Sikhism	1 (0.3)	0 (0.0)	1 (0.3)	0 (0.0)	1 (0.3)	0 (0.0)	1 (0.3)	0 (0.0)	1 (0.3)	0 (0.0)	1 (0.4)	0 (0.0)
Statistical analysis		χ2 = 1.340, df = 3, *P* = 0.720		χ2 = 1.524, df = 3, *P* = 0.677		χ2 = 1.386, df = 3, *P* = 0.709		χ2 = 1.447, df = 6, *P* = 0.963		χ2 = 9.620, df = 3, *P* = 0.22		χ2 = 7.350, df = 6, *P* = 0.290	
Ethnicity	Punjabi	198 (53.2)	41 (50.0)	207 (57)	32 (35.2)	215 (55.3)	24 (36.9)	177 (57.5)	62 (43.1)	200 (59.5)	39 (33.1)	166 (59.5)	66 (41.0)
	Sindhi	5 (1.3)	1 (1.2)	6 (1.7)	0 (0.0)	6 (1.5)	0 (0.0)	4 (1.3)	2 (1.4)	6 (1.8)	0 (0.0)	3 (1.1)	3 (1.9)
	Balochi	2 (2.4)	9 (2.4)	10 (2.8)	1 (1.1)	10 (2.6)	1 (1.5)	8 (2.6)	3 (2.1)	9 (2.7)	2 (1.7)	4 (1.4)	7 (4.3)
	Pathan	54 (14.5)	8 (9.8)	49 (13.5)	13 (14.3)	51 (13.1)	11 (16.9)	3 (11.7)	24 (16.7)	43 (12.8)	19 (16.1)	38 (13.6)	17 (10.6)
	Balti	3 (0.8)	3 (3.7)	5 (1.4)	1 (1.1)	6 (1.5)	0 (0.0)	6 (1.9)	0 (0.0)	3 (0.9)	3 (2.5)	3 (1.1)	3 (1.9)
	Kashmiri	13 (3.5)	0 (0.0)	13 (3.6)	0 (0.0)	12 (3.1)	1 (1.5)	11 (3.6)	2 (1.4)	10 (3.0)	3 (2.5)	9 (3.2)	4 (2.5)
	Others	90 (24.2)	27 (32.9)	73 (20.1)	44 (48.4)	89 (22.9)	28 (43.1)	66 (21.4)	51 (35.4)	65 (19.3)	52 (44.1)	56 (20.1)	61 (37.9)
Statistical analysis		χ2 = 10.245, df = 6, *P* = 0.115		χ2 = 34.785, df = 6, *P* = 0.000		χ2 = 15.685, df = 6, *P* = 0.016		χ2 = 30.367, df = 12, *P* = 0.002		χ2 = 37.347, df = 6, *P* = 0.000		χ2 = 43.508, df = 12, *P* = 0.000	
Education	No formal education	14 (3.8)	0 (0.0)	13 (3.6)	1 (1.1)	13 (3.3)	1 (1.5)	10 (3.2)	3 (2.1)	13 (3.9)	1 (0.8)	10 (3.6)	3 (1.9)
	Primary	11 (3.0)	5 (6.1)	12 (3.3)	4 (4.4)	14 (3.6)	2 (3.1)	8 (2.6)	8 (5.6)	13 (3.9)	3 (2.5)	8 (2.9)	8 (5.0)
	Secondary	14 (3.8)	7 (8.5)	13 (3.6)	8 (8.8)	16 (4.1)	5 (7.7)	8 (2.6)	13 (9.0)	6 (1.8)	15 (12.7)	2 (0.7)	19 (11.8)
	Post-secondary	333 (89.5)	70 (85.4)	325 (89.5)	78 (85.7)	346 (88.9)	57 (87.7)	282 (91.6)	120 (83.3)	304 (90.5)	99 (83.9)	259 (92.8)	131 (81.4)
Statistical analysis		χ2 = 8.406, df = 3, *P* = 0.038		χ2 = 6.088, df = 3, *P* = 0.107		χ2 = 2.183, df = 3, *P* = 0.535		χ2 = 27.230, df = 6, *P* = 0.000		χ2 = 25.987, df = 3, *P* = 0.000		χ2 = 32.865, df = 6, *P* = 0.000	
Marital status	Single	254 (68.3)	65 (79.3)	245 (67.5)	74 (81.3)	272 (69.9)	47 (72.3)	218 (70.8)	101 (70.1)	228 (67.9)	91 (77.1)	192 (68.8)	115 (71.4)
	Married	118 (31.7)	17 (20.7)	118 (32.5)	17 (18.7)	117 (30.1)	18 (27.7)	90 (29.2)	43 (29.9)	108 (32.1)	27 (22.9)	87 (31.2)	46 (28.6)
Statistical analysis		χ2 = 3.883, df = 1, *P* = 0.049		χ2 = 6.656, df = 1, *P* = 0.010		χ2 = 0.152, df = 1, *P* = 0.697		χ2 = 4.766, df = 2, *P* = 0.092		χ2 = 3.585, df = 1, *P* = 0.058		χ2 = 1.984, df = 2, *P* = 0.371	
Occupation	Butchers	0 (0.0)	1 (1.2)	0 (0.0)	1 (1.1)	0 (0.0)	1 (1.5)	0 (0.0)	1 (0.7)	0 (0.0)	1 (0.8)	1 (0.4)	0 (0.0)
	Farmers	25 (6.7)	9 (11.0)	29 (8.0)	5 (5.5)	30 (7.7)	4 (6.2)	24 (7.8)	9 (6.3)	28 (8.3)	6 (5.1)	19 (6.8)	14 (8.7)
	Livestock keepers	13 (3.5)	1 (1.2)	10 (2.8)	4 (4.4)	14 (3.6)	0 (0.0)	9 (2.9)	4 (2.8)	11 (3.3)	3 (2.5)	9 (3.2)	4 (2.5)
	Others	334 (89.8)	71 (86.6)	324 (89.3)	81 (89.0)	345 (88.7)	60 (92.3)	275 (89.3)	130 (90.3)	297 (88.4)	108 (91.5)	250 (89.6)	143 (88.8)
Statistical analysis		χ2 = 7.366, df = 3, *P* = 0.061		χ2 = 5.229, df = 3, *P* = 0.156		χ2 = 8.586, df = 3, *P* = 0.035		χ2 = 23.461, df = 6, *P* = 0.001		χ2 = 4.326, df = 3, *P* = 0.228		χ2 = 2.095, df = 6, *P* = 0.911	
Income	Below 30,000	51 (13.7)	11 (13.4)	47 (12.9)	15 (16.5)	47 (12.1)	15 (23.1)	39 (12.7)	22 (15.3)	48 (14.3)	14 (11.9)	38 (13.6)	23 (14.3)
	31,000-60,000	101 (27.2)	20 (24.4)	97 (26.7)	24 (26.4)	102 (26.2)	19 (29.2)	83 (26.9)	37 (25.7)	98 (29.2)	23 (19.5)	72 (25.8)	45 (28.0)
	61,000-90,000	74 (19.9)	17 (20.7)	73 (20.1)	18 (19.8)	82 (21.1)	9 (13.8)	65 (21.1)	26 (18.1)	69 (20.5)	22 (18.6)	60 (21.5)	28 (17.4)
	Above 90,000	146 (39.2)	34 (41.5)	146 (40.2)	34 (37.4)	158 (40.6)	22 (33.8)	121 (39.3)	59 (41.0)	121 (36.0)	59 (50.0)	109 (39.1)	65 (40.4)
Statistical analysis		χ2 = 0.303, df = 3, *P* = 0.959		χ2 = 0.823, df = 3, *P* = 0.844		χ2 = 7.217, df = 3, *P* = 0.065		χ2 = 4.628, df = 6, *P* = 0.592		χ2 = 7.908, df = 3, *P* = 0.048		χ2 = 1.635, df = 6, *P* = 0.950	

*Q1, Do you think that social, political, economic instability contributes to spread of CE?*

*Q2, Do you think that unchecked, unhygienic systems of slaughtering and animal keeping contributes toward prevalence of CE?*

*Q3, Do you think that lack of awareness is one of the potential risk factor of echinococcosis?*

*Q4, Do exposure to dog feces may lead to get infected with CE infection?*

*Q5, Do contaminated food/ water consumption may cause echinococcosis?*

*Q6, Do you think CE is asymptomatic disease or not?*

**Table 8:** Presentation of Community perception across sociodemographic factors.

**Sociodemographic factors**		**Community perception (%)**																			
**Variables**	**Features**	**Q1**		**Q2**		**Q3**		**Q4**		**Q5**		**Q6**		**Q7**		**Q8**		**Q9**		**Q10**	
		**F**	**A**	**F**	**A**	**F**	**A**	**F**	**A**	**F**	**A**	**F**	**A**	**F**	**A**	**F**	**A**	**F**	**A**	**F**	**A**
Age (years)	15–30	47 (60.3)	278 (73.9)	151 (69.9)	174 (73.1)	180 (70.9)	145 (72.5)	221 (74.2)	104 (66.7)	164 (69.8)	161 (73.5)	207 (70.9)	118 (72.8)	169 (69.8)	156 (73.6)	220 (68.1)	105 (80.2)	202 (72.9)	123 (69.5)	216 (75.8)	109 (64.5)
	31–45	18 (23.1)	65 (17.3)	43 (19.9)	40 (16.8)	51 (20.1)	32 (16)	50 (16.8)	33 (21.2)	46 (19.6)	37 (16.9)	59 (20.2)	24 (14.8)	43 (17.8)	40 (18.9)	65 (20.1)	18 (13.7)	48 (17.3)	35 (19.8)	49 (17.2)	34 (20.1)
	46–60	11 (14.1)	26 (6.9)	21 (9.7)	16 (6.7)	17 (6.7)	20 (10)	20 (6.7)	17 (10.9)	20 (8.5)	17 (7.8)	21 (7.2)	16 (9.9)	27 (11.2)	10 (4.7)	30 (9.3)	7 (5.3)	20 (7.2)	17 (9.6)	16 (5.6)	21 (12.4)
	61–75	2 (2.6)	7 (1.9)	1 (0.5)	8 (3.4)	6 (2.4)	3 (1.5)	7 (2.3)	2 (1.3)	5 (2.1)	4 (1.8)	5 (1.7)	4 (2.5)	3 (1.2)	6 (2.8)	8 (2.5)	1 (0.8)	7 (2.5)	2 (1.1)	4 (1.4)	5 (3.0)
Statistical analysis		χ2 = 7.129, df = 3, P = 0.68		χ2 = 6.806, df = 3, P = 0.78		χ2 = 2.981, df = 3, P = 0.395		χ2 = 4.665, df = 3, P = 0.198		χ2 = 0.795, df = 3, P = 0.851		χ2 = 2.934, df = 3, P = 0.402		χ2 = 7.490, df = 3, P = 0.058		χ2 = 7.124, df = 3, P = 0.068		χ2 = 2.348, df = 3, P = 0.503		χ2 = 9.721, df = 3, P = 0.021	
Sex	Male	38 (48.7)	160 (42.6)	87 (40.3)	111 (46.6)	94 (37.0)	104 (52.0)	124 (41.6)	74 (47.4)	106 (45.1)	92 (42.0)	129 (44.2)	69 (42.6)	110 (45.5)	88 (41.5)	142 (44)	56 (42.7)	122 (44)	76 (42.9)	123 (43.2)	75 (44.4)
	Female	40 (51.3)	216 (57.4)	129 (59.7)	127 (53.4)	160 (63.0)	96 (48.0)	174 (58.4)	82 (52.6)	129 (54.9)	127 (58.8)	163 (55.8)	93 (57.4)	132 (54.5)	124 (58.5)	181 (56)	75 (57.3)	155 (56)	101 (57.1)	162 (56.8)	94 (55.6)
Statistical analysis		χ2 = 0.998, df = 1, P = 0.318		χ2 = 1.863, df = 1, P = 0.172		χ2 = 10.227, df = 1, P = 0.001		χ2 = 1.413, df = 1, P = 0.235		χ2 = 0.442, df = 1, P = 0.506		χ2 = 0.107, df = 1, P = 0.744		χ2 = 715, df = 1, P = 0.398		χ2 = 0.056, df = 1, P = 0.813		χ2 = 0.054, df = 1, P = 0.817		χ2 = 064, df = 1, P = 0.800	
Religion	Islam	76 (97.4)	372 (98.9)	215 (99.5)	233 (97.9)	238 (100)	194 (97)	295 (99.9)	153 (98.1)	229 (97.4)	219 (100)	288 (98.6)	160 (98.8)	241 (99.6)	207 (97.6)	319 (98.8)	129 (98.5)	274 (98.9)	174 (98.3)	281 (98.6)	167 (98.8)
	Christianity	2 (2.6)	1 (0.3)	0 (0.0)	3 (1.3)	0 (0.0)	3 (1.5)	2 (0.7)	1 (0.6)	3 (1.3)	0 (0.0)	3 (1.0)	0 (0.0)	1 (0.4)	2 (0.9)	1 (0.3)	2 (1.5)	1 (0.4)	2 (1.1)	1 (0.4)	2 (1.2)
	Hindu	0 (0.0)	2 (0.5)	1 (0.5)	1 (0.4)	0 (0.0)	2 (1)	1 (0.3)	1 (0.6)	2 (0.9)	0 (0.0)	1 (0.3)	1 (0.6)	0 (0.0)	2 (0.9)	2 (0.6)	0 (0.0)	1 (0.4)	1 (0.6)	2 (0.7)	0 (0.0)
	Sikhism	0 (0.0)	1 (0.3)	0 (0.0)	1 (0.4)	0 (0.0)	1 (0.5)	0 (0.0)	1 (0.6)	1 (0.4)	0 (0.0)	0 (0.0)	1 (0.6)	0 (0.0)	1 (0.5)	1 (0.3)	0 (0.0)	1 (0.4)	0 (0.0)	1 (0.4)	0 (0.0)
Statistical analysis		χ2 = 5.800, df = 3, P = 0.122		χ2 = 3.666, df = 3, P = 0.300		χ2 = 7.722, df = 3, P = 0.052		χ2 = 2.173, df = 3, P = 0.544		χ2 = 5.666, df = 3, P = 0.129		χ2 = 3.646, df = 3, P = 0.302		χ2 = 3.949, df = 3, P = 0.267		χ2 = 3.307, df = 3, P = 0.347		χ2 = 1.711, df = 3, P = 0.634		χ2 = 2.892, df = 3, P = 0.409	
Ethnicity	Punjabi	40 (51.3)	199 (52.9)	117 (54.2)	122 (51.3)	137 (53.9)	102 (51.0)	158 (53.0)	81 (51.9)	114 (48.5)	125 (57.1)	159 (54.5)	80 (49.4)	124 (51.2)	115 (54.2)	181 (56.0)	58 (44.3)	147 (53.1)	92 (52.0)	145 (50.9)	94 (55.6)
	Sindhi	1 (1.3)	5 (1.3)	3 (1.4)	3 (1.3)	6 (2.4)	0 (0.0)	5 (1.7)	1 (0.6)	4 (1.7)	2 (0.9)	5 (1.7)	1 (0.6)	3 (1.2)	3 (1.4)	5 (1.5)	1 (0.8)	4 (1.4)	2 (1.1)	2 (0.7)	4 (2.4)
	Balochi	1 (1.3)	10 (2.7)	4 (1.9)	7 (2.9)	5 (2.0)	6 (3.0)	5 (1.7)	6 (3.8)	5 (2.1)	6 (2.7)	6 (2.1)	5 (3.1)	9 (3.7)	2 (0.9)	9 (2.8)	2 (1.5)	6 (2.2)	5 (2.8)	6 (2.1)	5 (3.0)
	Pathan	12 (15.4)	50 (13.3)	29 (13.4)	33 (13.9)	26 (10.2)	36 (18.0)	37 (12.4)	25 (16.0)	30 (12.8)	32 (14.6)	37 (12.7)	25 (15.4)	36 (14.9)	26 (12.3)	43 (13.3)	19 (14.5)	35 (12.6)	27 (15.3)	31 (10.9)	31 (18.3)
	Balti	4 (5.1)	2 (0.5)	0 (0.0)	6 (2.5)	3 (1.2)	3 (1.5)	4 (1.3)	2 (1.3)	5 (2.1)	1 (0.5)	6 (2.1)	0 (0.0)	5 (2.1)	1 (0.5)	3 (0.9)	3 (2.3)	4 (1.4)	2 (1.1)	6 (2.1)	0 (0.0)
	Kashmiri	3 (3.8)	10 (2.7)	2 (0.9)	11 (4.6)	8 (3.1)	5 (2.5)	8 (2.7)	5 (3.2)	8 (3.4)	5 (2.3)	9 (3.1)	4 (2.5)	3 (1.2)	10 (4.7)	8 (2.5)	5 (3.8)	5 (1.8)	8 (4.5)	7 (2.5)	6 (3.6)
	Others	17 (21.8)	100 (26.6)	61 (28.2)	56 (23.5)	69 (27.2)	48 (24.0)	81 (27.2)	36 (23.1)	69 (29.4)	48 (21.9)	70 (24.0)	47 (29.0)	62 (25.6)	55 (25.9)	74 (22.9)	43 (32.8)	76 (27.4)	41 (23.2)	88 (30.9)	29 (17.2)
Statistical analysis		χ2 = 11.968, df = 6, P = 0.063		χ2 = 12.589, df = 6, P = 0.050		χ2 = 11.024, df = 6, P = 0.088		χ2 = 4.589, df = 6, P = 0.597		χ2 = 7.903, df = 6, P = 0.245		χ2 = 6.986, df = 6, P = 0.322		χ2 = 11.328, df = 6, P = 0.079		χ2 = 9.037, df = 6, P = 0.172		χ2 = 4.466, df = 6, P = 0.614		χ2 = 19.076, df = 6, P = 0.004	
Education	No formal education	4 (5.1)	10 (2.7)	9 (4.2)	5 (2.1)	9 (3.5)	5 (2.5)	9 (3.0)	5 (3.2)	6 (2.6)	8 (3.7)	6 (2.1)	8 (4.9)	9 (3.7)	5 (2.4)	10 (3.1)	4 (3.1)	7 (2.5)	7 (4.0)	3 (1.1)	11 (6.5)
	Primary	4 (5.1)	12 (3.2)	8 (3.7)	8 (3.4)	10 (3.9)	6 (3.0)	10 (3.4)	6 (3.8)	9 (3.8)	7 (3.2)	11 (3.8)	5 (3.1)	11 (4.5)	5 (2.4)	15 (4.6)	1 (0.8)	7 (2.5)	9 (5.1)	7 (2.5)	9 (5.3)
	Secondary	4 (5.1)	17 (4.5)	8 (3.7)	13 (5.5)	8 (3.1)	13 (6.5)	14 (4.7)	7 (4.5)	13 (5.5)	8 (3.7)	12 (4.1)	9 (5.6)	8 (3.3)	13 (6.1)	10 (3.1)	11 (8.4)	15 (5.4)	6 (3.4)	14 (4.9)	7 (4.1)
	Post-Secondary	66 (84.6)	337 (89.6)	191 (88.4)	212 (89.1)	227 (89.4)	176 (88.0)	265 (88.9)	138 (88.5)	207 (88.1)	196 (89.5)	263 (90.1)	140 (86.4)	214 (88.4)	189 (89.2)	288 (89.2)	115 (87.8)	248 (89.5)	155 (87.6)	261 (91.6)	142 (84.0)
Statistical analysis		χ2 = 2.198, df = 3, P = 0.532		χ2 = 2.367, df = 3, P = 0.500		χ2 = 3.413, df = 3, P = 0.332		χ2 = 0.094, df = 3, P = 0.993		χ2 = 1.464, df = 3, P = 0.691		χ2 = 3.574, df = 3, P = 0.311		χ2 = 4.170, df = 3, P = 0.244		χ2 = 0.9665 df = 3, P = 0.022		χ2 = 3.723, df = 3, P = 0.293		χ2 = 13.539, df = 3, P = 0.004	
Marital status	Single	46 (59.0)	273 (72.6)	142 65.7)	177 (74.4)	170 (66.9)	149 (74.5)	216 (72.5)	103 (66.0)	160 (68.1)	159 (72.6)	200 (68.5)	119 (73.5)	166 (68.6)	153 (72.2)	214 (66.3)	105 (80.2)	191 (69.0)	128 (72.3)	211 (74.0)	108 (63.9)
	Married	32 (41.0)	103 (27.4)	74 34.3)	61 (25.6)	84 (33.1)	51 (25.5)	82 (27.5)	53 (34.0)	75 (31.9)	60 (27.4)	92 (31.5)	43 (26.5)	76 (31.4)	59 (27.8)	109 (33.7)	26 (19.8)	86 (31.0)	49 (27.7)	74 (26.0)	61 (36.1)
Statistical analysis		χ2 = 5.746, df = 1, P = 0.017		χ2 = 4.035, df = 1, P = 0.045		χ2 = 3.070, df = 1, P = 0.80		χ2 = 2.044, df = 1, P = 0.153		χ2 = 1.107, df = 1, P = 0.293		χ2 = 1.229, df = 1, P = 0.268		χ2 = 0.691, df = 1, P = 0.406		χ2 = 8.617, df = 1, P = 0.003		χ2 = 0.585, df = 1, P = 0.444		χ2 = 5.210, df = 1, P = 0.022	
Occupation	Butchers	0 (0.0)	1 (0.3)	1 (0.5)	0 (0.0)	0 (0.0)	1 (0.5)	0 (0.0)	1 (0.6)	0 (0.0)	1 (0.5)	1 (0.3)	0 (0.0)	0 (0.0)	1 (0.5)	0 (0.0)	1 (0.8)	0 (0.0)	1 (0.6)	0 (0.0)	1 (0.6)
	Farmers	10 (12.8)	24 (6.4)	15 (6.9)	19 (8.0)	19 (7.5)	15 (7.5)	16 (5.4)	18 (11.5)	19 (8.1)	15 (6.8)	20 (6.8)	14 (8.6)	19 (7.9)	15 (7.1)	27 (8.4)	7 (5.3)	22 (7.9)	12 (6.8)	19 (6.7)	15 (8.9)
	Livestock Keepers	2 (2.6)	12 (3.2)	5 (2.3)	9 (3.8)	11 (4.3)	3 (1.5)	12 (4.0)	2 (1.3)	9 (3.8)	5 (2.3)	7 (2.4)	7 (4.3)	7 (2.9)	7 (3.3)	9 (2.8)	5 (3.8)	9 (3.2)	5 (2.8)	9 (3.2)	5 (3.0)
	Others	66 (84.6)	339 (90.2)	195 (90.3)	210 (88.2)	224 (88.2)	181 (90.5)	270 (90.6)	135 (86.5)	207 (88.1)	198 (90.4)	264 (90.4)	141 (87.0)	216 (89.3)	189 (89.2)	287 (89.9)	118 (90.1)	246 (88.8)	159 (89.8)	257 (90.2)	148 (87.6)
Statistical analysis		χ2 = 4.087, df = 3, P = 0.252		χ2 = 2.108, df = 3, P = 0.550		χ2 = 4.245, df = 3, P = 0.236		χ2 = 9.806, df = 3, P = 0.020		χ2 = 2.252, df = 3, P = 0.522		χ2 = 2.385, df = 3, P = 0.496		χ2 = 1.294, df = 3, P = 0.731		χ2 = 3.934, df = 3, P = 0.269		χ2 = 1.836, df = 3, P = 0.607		χ2 = 2.472, df = 3, P = 0.480	
Income	Below 30,000	16 (20.5)	46 (12.2)	38 17.6)	24 (10.1)	34 (13.4)	28 (14.0)	37 (12.4)	25 (16.0)	30 (12.8)	32 (14.6)	39 (13.4)	23 (14.2)	41 (16.9)	21 (9.9)	48 (14.9)	14 (10.7)	41 (14.8)	21 (11.9)	43 (15.1)	19 (11.2)
	31,000–60,000	15 (19.2)	106 (28.2)	57 26.4)	64 (26.9)	67 (26.4)	54 (27.0)	79 (26.5)	42 (26.9)	58 (24.7)	63 (28.8)	81 (27.7)	40 (24.7)	64 (26.4)	57 (26.9)	87 (26.9)	34 (26.0)	77 (27.8)	44 (24.9)	73 (25.6)	48 (28.4)
	61,000–90,000	17 (21.8)	74 (19.7)	40 18.5)	51 (21.4)	53 (20.9)	38 (19.0)	61 (20.5)	30 (19.2)	46 (19.6)	45 (20.5)	58 (19.9)	33 (20.4)	49 (20.2)	42 (19.8)	59 (18.3)	32 (24.4)	50 (18.1)	41 (23.2)	53 (18.6)	38 (22.5)
	Above 90,000	30 38.5)	150 (39.9)	81 37.5)	99 (41.6)	100 (39.4)	80 (40.0)	121 (40.6)	59 (37.8)	101 (43.0)	79 (36.1)	114 (39.0)	66 (40.7)	89 (36.4)	92 (43.4)	129 (39.9)	51 (38.9)	109 (39.4)	71 (40.1)	116 (40.7)	64 (37.9)
Statistical analysis		χ2 = 5.366, df = 3, P = 0.147		χ2 = 5.643, df = 3, P = 0.130		χ2 = 0.253, df = 3, P = 0.969		χ2 = 1.262, df = 3, P = 0.738		χ2 = 2.410, df = 3, P = 0.492		χ2 = 0.507, df = 3, P = 0.917		χ2 = 5.526, df = 3, P = 0.137		χ2 = 3.011, df = 3, P = 0.390		χ2 = 2.457, df = 3, P = 0.483		χ2 = 2.473, df = 3, P = 0.480	

*Q1, Should we kill all dogs?*

*Q2, Should we kill stray dogs only?*

*Q3, Should we stop feeding dogs with sheep cysts?*

*Q4, Should we feed dogs personally?*

*Q5, Prevention is better than treatment?*

*Q6, Should we bury/burn infected organs?*

*Q7, Should we stop owning dogs?*

*Q8, Should we stop throwing away carcasses?*

*Q9, Should we replace sheep with goats?*

*Q10, Should we reduce dogs' access to slaughter areas?*

### Statistical Analysis for Knowledge, Attitude, Practices, One Health Concept, Risk Factors, and Community Perception of Cystic Echinococcosis

The factors that determine views on KAP, One Health, risk factors, and community perception (dependent variables) about CE included age, sex, religion, ethnicity, education, marital status, occupation, and income (independent variables). The results of the analysis demonstrated that only ethnicity (*p* < 0.05) within a section of knowledge was significant, while sex (*p* = 0.05) was close to significance. Among practices, significant differences were only demonstrated for sex (*p* < 0.05; Tables 9, 10).

**Table 9:** Statistical analysis of knowledge, attitude, and practices across different socio-demographic variables.

**Factors**	**Knowledge score** **(out of 9)**		**Attitudes score** **(out of 5)**		**Practices score** **(out of 16)**	
	**Mean (SD)**	***P-*value**	**Mean (SD)**	***P-*value**	**Mean (SD)**	***P-*value**
Age (years)						
15–30	2.31 (1.64)	0.945	2.62 (1.45)	0.108	6.65 (2.68)	0.589
31–45	2.34 (1.84)		2.45 (1.45)		6.41 (3.26)	
46–60	2.23 (1.73)		2.07 (1.46)		5.97 (1.76)	
61–75	2.63 (0.92)		3.25 (1.39)		6.88 (2.9)	
Sex						
Female	2.52 (1.67)	0.0544	2.7 (1.5)	0.0826	6.18 (2.54)	0.0408
Male	2.14 (1.67)		2.4 (1.41)		6.84 (2.91)	
Religion						
Muslim	2.31 (1.68)	0.556	2.55 (1.47)	0.907	6.54 (2.77)	0.628
Christian	4.00 (0.00)		2.00 (0.00)		7.00 (0.00)	
Hindu	2.00 (NA)		2.00 (NA)		4.00 (NA)	
Sikh	2.00 (NA)		2.00 (NA)		4.00 (NA)	
Ethnicity						
Punjabi	2.34 (1.74)	0.0378	2.56 (1.49)	0.136	6.27 (2.79)	0.275
Pathan	1.94 (1.45)		2.14 (1.51)		7.37 (3.18)	
Kashmiri	3.00 (1.67)		2.55 (1.21)		6.82 (1.66)	
Baloch	2.36 (1.63)		3.09 (1.30)		6.55 (1.7)	
Sindhi	1.83 (1.72)		2.33 (0.82)		6.50 (1.23)	
Hazaragi	0.00 (0.00)		4.00 (0.00)		7.00 (0.58)	
Balti	2.86 (0.69)		2.57 (0.79)		7.57 (7.57)	
Hunza people	4.00 (1.00)		4.00 (0.00)		8.67 (NA)	
Afghani	6.00 (NA)		1.00 (NA)		6.00 (NA)	
Urdu speaking	2.67 (0.82)		3.33 (1.03)		5.17 (1.72)	
Education						
Post-secondary	2.28 (1.67)	0.31	2.58 (1.47)	0.578	6.58 (2.89)	0.732
Secondary level	2.55 (1.78)		2.24 (1.43)		6.62 (1.86)	
Primary level	1.93 (1.59)		2.29 (1.49)		5.93 (2.59)	
No formal education	3.10 (1.66)		2.70 (1.16)		5.90 (1.85)	
Marital Status						
Single	2.36 (1.69)	0.569	2.64 (1.40)	0.0934	6.63 (2.81)	0.378
Married	2.25 (1.66)		2.35 (1.54)		6.34 (2.67)	
Occupation						
Butchers	3.44 (2.60)	0.197	2.44 (1.59)	0.594	6.89 (2.80)	0.623
	2.44 (1.65)		2.61 (1.29)		5.83 (1.82)	
Keepers	2.67 (1.37)		3.33 (0.82)		5.83 (3.66)	
Other professions	2.27 (1.64)		2.52 (1.48)		6.58 (2.79)	
Income						
30,000 & below	2.34 (1.64)	0.162	2.28 (1.42)	0.582	6.59 (2.09)	0.156
31,000–60,000	2.61 (1.87)		2.59 (1.5)		6.9 (2.67)	
61,000–90,000	2.01 (1.65)		2.45 (1.54)		5.93 (2.74)	
90,000 above	2.3 (1.53)		2.65 (1.38)		1.65 (2.97)	

**Table 10:** Statistical analysis of one health concept, risk factors, and community perceptions.

**Factors**	**One Health Concept score (out of 7)**		**Risk Factors score** **(out of 6)**		**Community Perception score (out of 10)**	
	**Mean (SD)**	***p*-value**	**Mean (SD)**	***p*-value**	**Mean (SD)**	***p*-value**
**Age (years)**						
15–30	6.60 (1.02)	0.872	5.01 (1.41)	0.813	5.4 (1.89)	0.605
31–45	6.49 (1.34)		4.82 (1.46)		5.71 (1.82)	
46–60	6.55 (1.21)		5.00 (1.59)		5.48 (1.79)	
61–75	6.75 (0.71)		4.88 (1.46)		5.88 (1.46)	
**Sex**						
Female	6.57 (1.23)	0.987	5.09 (1.37)	0.134	5.60 (1.87)	0.35
Male	6.57 (1.003)		4.84 (1.5)		5.40 (1.83)	
**Religion**						
Muslim	6.57 (1.12)	0.845	4.96 (1.45)	0.588	5.52 (1.85)	0.449
Christian	6.00 (0.00)		4.00 (0.00)		4.00 (0.00)	
Hindu	7.00 (NA)		6.00 (NA)		4.00 (NA)	
Sikh	7.00 (NA)		6.00 (NA)		4.00 (NA)	
**Ethnicity**						
Punjabi	6.55 (1.18)	0.177	5.05 (1.44)	0.355	5.55 (1.92)	0.31
Pathan	6.67 (0.93)		4.57 (1.57)		5.24 (1.81)	
Kashmiri	6.73 (0.47)		5.18 (0.98)		4.73 (0.91)	
Baloch	6.55 (1.21)		4.55 (1.37)		5.09 (1.58)	
Sindhi	7.00 (0.00)		5.00 (1.1)		6.33 (1.03)	
Hazaragi	7.00 (0.00)		6.00 (0.00)		6.00 (0.00)	
Balti	5.29 (1.60)		4.29 (1.70)		6.00 (2.31)	
Hunza people	7.00 (0.00)		5.67 (0.58)		5.00 (0.00)	
Afghani	7.00 (NA)		5.00 (NA)		9.00 (NA)	
Urdu speaking	7.00 (0)		5.50 (1.23)		6.33 (NA)	
**Education**						
Post-secondary	6.59 (1.12)	0.371	5.00 (1.41)	0.163	5.52 (1.85)	0.732
Secondary level	6.48 (1.09)		4.83 (1.49)		5.35 (1.93)	
Primary level	6.214 (1.48)		4.21 (1.89)		5.79 (1.85)	
No formal education	7.00 (0.00)		5.40 (0.97)		5.00 (1.63)	
**Marital Status**						
Single	6.62 (1.01)	0.29	4.98 (1.43)	0.753	5.36 (1.84)	0.085
Married	6.48 (1.29)		4.93 (1.47)		5.75 (1.84)	
**Occupation**						
Butchers	7.00 (0.00)	0.479	5.33 (1.00)	0.627	5.11 (1.83)	0.505
Keepers	6.44 (1.2)		4.67 (1.61)		5.67 (1.5)	
Farmers	7.00 (0.00)		5.33 (0.82)		6.50 (1.64)	
Other professions	6.56 (1.14)		4.96 (1.45)		5.48 (1.87)	
**Income**						
30,000 & below	6.63 (1.01)	0.419	4.94 (1.48)	0.945	6.03 (1.45)	0.235
31,000–60,000	6.73 (0.93)		5.00 (1.4)		5.59 (1.91)	
61,000–90,000	6.53 (1.14)		5.01 (1.47)		5.46 (1.88)	
90,000 above	6.47 (1.25)		4.9 (1.46)		5.29 (1.87)	

## Discussion

### The Sociodemographic Background of the Participants

Age, sex, religion, ethnicity, education, marital status, occupation, and income were included as key sociodemographic factors in the analysis to examine their role and association with the spread of CE in Pakistan. A previous study has shown that moving from high to low on any socioeconomic aspect, income, or education results in a decline in health (21).

More highly educated individuals reported better health and lower mortality risks in various studies conducted in different countries (25–27). Among 454 surveyed individuals in the current study, very few people were illiterate (3.1%; 14/454), while 3.5% (16/454) had experienced primary education and 4.6% (21/454) had secondary level education. Most, 88.8% (403/454), had post-secondary education. All participants had little knowledge of zoonotic infections. Participants showed no significant difference in their level of knowledge, despite belonging to varying educational backgrounds. This scenario is not unusual for neglected tropical diseases such as CE, where even the educated population is unaware of the disease. However, education does have a role in other factors associated with CE, such as hand washing, water boiling, and perception of the disease. This highlights the role of education in the control of disease transmission and better education is expected to reduce transmission of the disease (28).

Many public health researchers state that income and associated resources are the most influential factors on health and mortality (29). In one study, an increase in household income from $20,000 to $70,000 reduced the odds of mortality by 50% (30). An interesting finding in our study was that participants were reluctant to seek treatment for financial reasons.

Much research has linked health to occupational rank. Higher mortality rates in servants with low prestige jobs were associated with daily practices and behaviors, whereas officials with a higher rank had a lower obesity rate, fewer were addicted to tobacco, and they had a good diet, better health care practices, and less stress (31, 32). We observed similar findings: people belonging to livestock-related occupations, where they have contact with animals, such as butchers, keepers, and farmers, have higher chances of getting CE because of their occupation. Many other studies have reported the same observation, with a higher incidence of CE in a pastoral community in than participants with other occupations (33–36). In a survey among hunters in China, few cases were found (37). This low disease frequency in hunters and higher frequency in farmers proves that occupational activity contributes to the disease, not only because of the association with animals, but by certain behaviors, attitudes, perceptions, and practices, such as hand washing.

Different ethnic groups were included in the study to ascertain whether there are certain cultural practices that could promote the transmission of the disease and make it more common in a particular group. The results of suggested that there were no significant differences, indicating that CE is equally prevalent in all ethnic groups. This could be because, in Pakistan, the livestock-related practices are similar in all regions.

### KAP Analysis of CE

KAP questions were included to better analyse the major variables (risk factors, One Health, community perception) among participants, prompting them to link, compare, and evaluate these variables based on their knowledge and attitude toward the disease and the practices they follow.

The results of our survey corresponded well with those from one performed in 2018 in Pakistan (16), with participants in both studies demonstrating a clear lack of knowledge. Frequencies of familiarity with zoonoses now and in 2018 were 31.9 and 30%, respectively, while awareness of CE was 20.5 and 4.2%, indicating contrasting results compared with those of the previous study.

The participants were not familiar with the disease and its mode of transmission; therefore, they did not consider themselves at risk of developing the disease or getting infected by animals or other people.

A mixture of good and bad practices were found in both studies, based primarily on knowledge and perception of health in general and not about CE in particular. The frequency of people washing their hands before eating (90%) and after handling cattle (85%) demonstrated the general awareness of the population about cleanliness and the belief that not following these practices might affect their health and lifestyle. However, some specific practices, such as inspection of meat before consumption, either by themselves or by their animals (32%−2018, 63%–current) and the presence of stray dogs (70%−2018, 58%–current) demonstrated that people are not aware of the disease being spread by animals.

### The One Health Concept of CE

The concept that human health is linked to that of animals and the environment was well-known to the participants. Reservoirs of *Echinococcus* species and an increase in disease transmission are outcomes of urbanization. Anthropogenic environmental changes, such as those caused by deforestation and urbanization, affect wildlife and can lead to zoonotic disease transmission in humans (38).

Increased host range and enhanced parasitic transmission between definitive and intermediate hosts, caused by environmental changes, might put humans at risk of increased echinococcosis transmission (38). Intermediate hosts of zoonotic diseases feed on vegetation, and their numbers will increase because of improvements in the quality and quantity of their food source, thus increasing the potential for disease spread to humans (39–42).

Natural or anthropogenic ecological changes for migratory host species, such as lack of food, deforestation, and urbanization, have adverse effects on host migratory behaviors, and thus preventing migration would have a substantial impact on the transmission of species causing echinococcosis. Less or no migration would end up concentrating the entire population of wildlife in human settlements and would create competition for resources for their survival, ultimately transmitting the disease. For example, in Australia, deforestation was reported as the main cause of an outbreak of Hendra virus, a result of migration of flying foxes from forested areas to human settlements in search of food, thus transmitting the virus (43).

### Assessment of Risk Factors for CE

As mentioned earlier, the economy of Pakistan is highly dependent on livestock. This population is at high risk of developing CE because no proper hygiene practices are followed while dealing with animals (22). Most of the participants in this study also saw this as a major risk factor of CE in Pakistan.

Similar findings were recorded from rural households in Algeria. More cases were recorded from rural communities (71%), the reason being their dependence on livestock for the earnings, without awareness of prevention and curative measures. The association of disease with livestock contact was described in a study where the population associated with animal husbandry was more at risk of disease, with at least one case of CE per house in Algeria. A study from Algeria also recorded at least one case (*p* < 0.001) in 14.6% of rural and 4.6% of urban houses, suggesting that migration and urbanization are also risk factors, carrying the disease to the cities (44).

The socioeconomic and political situation is a risk factor for promoting this disease, similar to China (45). The same factor may also be a major reason for disease spread in Pakistan, as no policies, budget support for health issues, education, or other awareness programs exist to control the disease.

Human CE infection is mainly associated with contact with dogs. In a study in Algeria, 29.8% of participants reported that dogs had access to their homes and 9.3% reported access to kitchens, posing a major threat of disease transmission via food contamination with dog feces. Owning more than one dog was associated with hydatidosis (*p* < 0.1) in urban areas in Algeria. Moreover, the disease was also reported to be caused by the presence of stray dogs in the district (44). The scenario in Pakistan is similar, as the population of stray dogs is uncontrolled; therefore, human settlements are likely to be contaminated with their feces. Poor community awareness of CE and knowledge about modes of transmission were stated to be major risks in the present study. Similarly, in a study of CE in Algeria, disease awareness was about 50%, with only 21% of respondents aware of disease transmission from dogs to other animals and humans (44). Many other studies have reported similar findings, such as those in Morocco (46–49).

### Community Perception Toward CE

In the current study, several control measures related to CE were highlighted to ascertain how aware the participants were of the disease. Participants had mixed or ambiguous views, showing a lack of awareness about the disease: most of them (71%) disagreed with throwing carcasses in open areas, but 48% stated that prevention should not be preferred over treatment because it needs a high level of awareness. Almost 63% of them were in favor of keeping dogs away from slaughter areas; however, in Pakistan the slaughter areas are privately owned property and are not managed by government authorities. We concluded that in a country where people face health-related issues every day, they are willing to support any program promising to improve their living standard, but are not capable of identifying exactly what should be done.

A study in Morocco found that participants were willing to support suggested control measures, such as waste disposal systems, hygiene conditions, and management of slaughter areas (47). However, the specific cultural practices in the community, including offal and waste disposal systems, home slaughtering, and keeping animals are risk factors that hinder disease control. Despite acknowledging CE as a serious health risk, the community was not aware of the parasite, its life cycle, and other associated mechanisms. This highlights the significance of providing knowledge and awareness of the disease to the public (50).

## Conclusion

Our study demonstrated a low awareness of CE in Pakistan, despite the prevalence of the disease in the country. The population showed positive responses toward the treatment of the disease and to suggested risk factors and community perception aspects. Improving these could help to control the disease. The participants were unaware of the factors associated with the disease, such as its mode of transmission, practices favoring its spread, control, and other associated factors. However, the current practices being followed by them, directly or indirectly, can predispose them to parasitic infection and transmission of CE. The results of the present study add to the existing knowledge regarding KAPs of cystic echinococcosis One Health perspective and is the representative of a South Asian population (Pakistan). This study will also pave the way for further studies at national and international level. However, the current study also had some limitations such as the data from major cities may not present the clearer picture regarding CE in provinces or national level. Respondents with different origins, ages, ethnic groups, and professions should be equally participated in the study to provide deeper insights and true information about CE KAPs in Pakistan.

## Publisher Notes

Springer Nature remains neutral with regard to jurisdictional claims in published maps and institutional affiliations.

## Data Availability Statement

The original contributions generated for this study are included in the article/Supplementary Material, further inquiries can be directed to the corresponding author/s.

## Ethics Statement

The studies involving human participants were reviewed and approved by the Ethics Committee of COMSATS University (CIIT/Bio/ERB/21/01). Written informed consent to participate in this study was provided by the participants' legal guardian/next of kin.

## Author Contributions

AK and SAm collected the data and wrote the paper following discussions with MA, SN, and SS. HA and MK designed the study. DK, SAl, and WH helped in the analysis. RS, RA, JC, and AD-B revised the paper and improved the technical quality of the manuscript. HA supervised the study. All authors approved the final version of the paper.

## Conflict of Interest

The authors declare that the research was conducted in the absence of any commercial or financial relationships that could be construed as a potential conflict of interest.

## Abbreviations

WHO: World Health Organization
NZDs: Neglected Zoonotic Diseases
CE: Cystic Echinococcosis
DALYs: Disability Adjusted Life Years.
