# Supplementary material for: Community Based Assessment of Behavior and Awareness of Risk Factors of Cystic Echinococcosis in Major Cities of Pakistan: A One Health Perspective
Source: Front Public Health. 2021 Jun 4;9:648900. doi: 10.3389/fpubh.2021.648900 (PMC8213035; doi:10.3389/fpubh.2021.648900)
Supplement: Supplementary file 1 [file Data_Sheet_1.docx]

**Questionnaire: Community perception and awareness regarding risk factors of CE in Pakistan based on one health prospective**

**Socio-demography:**

1. District/Area/ Village__________
2. Birthplace ___________
3. Religion_____________
4. Ethnicity __________
5. Age____________
6. Sex  M  F
7. Marital status  Married  Unmarried
8. Education level

No formal education  Primary  Secondary  Post-Secondary

1. Occupation

Butcher  Farmers/Livestock  Animal keeper  Other Profession

1. Average monthly income of your household _________________

**Knowledge**: **Yes No**

1. Ever heard about Zoonotic disease?
2. Ever heard about Echinococcosis?
3. Ever been infected by this disease?
4. Any family member/s diagnosed with the disease?
5. Ever seen the disease in animal organs?
6. Ever seen the disease in man?
7. Will you separate an affected animal or person from the uninfected
8. Aware of the danger of eating food contaminated with dog’s faeces?
9. Do you know playing with dogs could get you infected with disease?

**Attitude** **Yes No**

1. Do you think you are at risk of developing Echinococcosis?
2. Do you think you might get infected by association with dogs/infected people?
3. Would you like to receive a disease inspection?
4. If you are suffering from the disease, would you take free treatment?
5. If you need surgery because of disease, would you like to undergo that?

**Practices: Yes No**

1. Do you have own dog(s)?
2. Are dogs fed slaughter wastes?
3. Do dogs have access to the slaughter areas?
4. Have your dog/s been dewormed or vaccinated?
5. Has your dog ever received veterinary care when sick?
6. Are dogs restricted/ leashed?
7. Do you have stray dogs in your village?
8. Do you or your children sometimes play with dogs?
9. Do you eat raw fruit, vegetables, and meat?
10. Do you boil water for drinking?
11. Do you wash your hands before eating food?
12. Do you wash hands after handling dogs?
13. Do you inspect meat at your homes/shops/slaughter areas?
14. Are slaughter areas clean and well managed?
15. Do you give dogs uncooked cattle/sheep organs to eat?
16. Any medical facilities/ treatment given to infected people?

**One Health Concept** **Yes No**

1. Do you agree the health of humans is linked to health of animals and  the environment?
2. Vaccination campaigns for animals and humans are required?
3. Proper treatment facilities needed?
4. Need for proper disposal and sewage systems?
5. The diet of people as well as animals should be inspected properly?
6. Awareness of the impact of the environment on humans and animals?
7. Economic stability to favour/ improve health?

**Risk Factors**  **Yes No**

1. Do you think that social, political, economic instability contributes to spread of CE?
2. Do you think that unchecked, unhygienic systems of slaughtering and animal keeping contributes towards prevalence of CE?
3. Do you think that lack of awareness is one of the potential risk factor of echinococcosis?
4. Do exposure to dog feces may lead to get infected with CE infection?
5. Do contaminated food/ water consumption may cause echinococcosis?
6. Do you thin CE is asymptomatic disease or not?

**Community Perception**

1. **Favour b. Against**
2. Kill all dogs?

Fewer problems  Dogs have a role (useful) and the right to live

1. Kill stray dogs only?

Help eliminate dogs and threats

Dogs always reappear (Reproduction difficult to control)

1. Stop feeding dogs with sheep cysts?

Contributes to decreasing the disease

Cysts can be found anywhere else (souk, slaughter areas)

1. Feeding dogs personally?

Prevent dogs going out to look for their food and returning with diseases

Impossible to educate a dog (big appetite)

1. Prevention versus treatment?

More efficient and less costly than treatment

Prevention needs high level of awareness and care

1. Bury/burn infected organs?

Prevents dogs’ access to organs

Dogs have a strong sense of smell and offal is not buried deep enough

1. Stop owning dogs?

Fewer problems  Dogs are needed

1. Stop throwing away carcasses?

Avoid the bad smell  Too costly and time consuming to bury them

1. Replace sheep with goats?

Healthier meat with fewer cysts found (less contact with dogs)

Against the culture (food habits, religion); many disadvantages of goat herding

1. Reduce dogs’ access to slaughter areas?

Dogs not exposed to intermediate hosts carrying parasite

The disease does not come from the slaughter areas but from the pastures
